# Comprehensive Genomic Characterization of *Cronobacter sakazakii* Isolates from Infant Formula Processing Facilities Using Whole-Genome Sequencing

**DOI:** 10.3390/microorganisms11112749

**Published:** 2023-11-11

**Authors:** Zeinab Ebrahimzadeh Mousavi, Leonard Koolman, Guerrino Macori, Séamus Fanning, Francis Butler

**Affiliations:** 1 UCD-Centre for Food Safety, School of Public Health, Physiotherapy and Sports Science, University College Dublin, D04 V1W8 Dublin, Ireland; leonard.koolman@ucd.ie (L.K.); guerrino.macori@ucd.ie (G.M.); sfanning@ucd.ie (S.F.); 2 School of Biosystems and Food Engineering, University College Dublin, D04 V1W8 Dublin, Ireland; f.butler@ucd.ie; 3 Department of Food Science and Engineering, Faculties of Agriculture and Natural Resources, University of Tehran, Karaj 6719418314, Iran

**Keywords:** *Cronobacter sakazakii*, whole-genome sequencing, antibiotic resistance, sequence type (ST), plasmids, virulence genes, public health

## Abstract

*Cronobacter sakazakii* is an opportunistic pathogen linked to outbreaks in powdered infant formula (PIF), primarily causing meningitis and necrotizing enterocolitis. Whole-genome sequencing (WGS) was used to characterize 18 *C. sakazakii* strains isolated from PIF (powdered infant formula) manufacturing plants (2011–2015). Sequence Type (ST) 1 was identified as the dominant sequence type, and all isolates carried virulence genes for chemotaxis, flagellar motion, and heat shock proteins. Multiple antibiotic resistance genes were detected, with all isolates exhibiting resistance to Cephalosporins and Tetracycline. A significant correlation existed between genotypic and phenotypic antibiotic resistance. The plasmid *Col(pHAD28)* was identified in the isolates recovered from the same PIF environment. All isolates harbored at least one intact phage. All the study isolates were compared with a collection of 96 publicly available *C. sakazakii* genomes to place these isolates within a global context. This comprehensive study, integrating phylogenetic, genomic, and epidemiological data, contributes to a deeper understanding of *Cronobacter* outbreaks. It provides valuable insights to enhance surveillance, prevention, and control strategies in food processing and public health contexts.

## 1. Introduction

*Cronobacter* spp. are Gram-negative opportunistic pathogens belonging to the family *Enterobacteriaceae*. The *Cronobacter* genus consists of seven species: *C. sakazakii*, *C. malonaticus*, *C. universalis*, *C. turicensis*, *C. muytjensii*, *C. dublinensis*, and *C. condiment* [1,2]. *C. sakazakii* and C. *malonaticus* have the most significant clinical importance and have been associated with outbreaks in PIF (powdered infant formula) [3,4]. Therefore, neonates and low-birth-weight infants are the most susceptible population group since they could become infected through the consumption of PIF, or through contaminated bottles and appliances used for PIF preparation. The clinical profiles are mainly severe infantile septicemia, necrotizing enterocolitis, meningitis, and serious neurological sequelae [1,2,5].

Due to the potential fatalities caused by the consumption of contaminated PIF in neonates and premature infants, there have been many food recalls due to PIF contamination with *Cronobacter* species in various countries [1,4,6,7,8,9]. Contamination of PIF with *Cronobacter* spp. and the management of risk to consumers is a challenge to public health, regulatory agencies, and manufacturers alike [10].

The pathogenicity and development of disease caused by *Cronobacter* depend on several key aspects such as virulence factors, which may be encoded by plasmids [4]. Various virulence traits have been identified so far, such as cell adhesion and invasion in cellular lines such as HEp-2 and Caco-2, flagellar motility, survival in macrophages, sialic acid utilization, capsule and endotoxin production, and the presence of virulence genes such as *ompA*, *cpa*, *fliC*, *hly*, *sip*, *aut*, *plas*, and *inv* [1,4,6]. In addition, antibiotic resistance is another aspect to be considered since *Cronobacter* resistance toward ß-lactam antibiotics such as the first and second generations of cephalosporins and ampicillin and the presence of resistance genes such as *marA*, *glpT*, *ampH*, *blaCSA*, and *mcr* in *Cronobacter* have been well documented [4,11,12]. The antibiotic resistance profile of *Cronobacter* spp. has paramount importance since rapid treatment of infected newborns is generally accomplished through antibiotic therapy [11].

The analysis of the complete genome of the *Cronobacter* strains isolated from PIF manufacturing plants enables us to identify new strains, taxonomic relationships, and genes associated with virulence and antibiotic resistance [5]. In recent years, WGS (whole-genome sequencing) has enabled researchers to perform extensive profiling and genotyping, such as multilocus sequence typing (MLST), and complete analysis of the pathogenesis process of *C. sakazakii* isolated from PIF plants is therefore possible. Consequently, more precise epidemiological links could be established [1,2,4].

The aim of this study was to perform a molecular characterization of 18 *C. sakazakii* strains recovered from PIF, raw materials, and environmental samples from PIF manufacturing facilities in Europe during the period 2011–2015 to genotype the isolates and identify the genes associated with virulence and antibiotic resistance, as well as plasmids and phages. In addition, all study isolates were compared in the context of global isolates to reveal possible epidemiological links.

## 2. Materials and Methods

### 2.1. Bacterial Isolates and Sample Preparation

Strains of *C. sakazakii* (n = 18) were maintained in cryogenic vials filled with stock culture beads at −80 °C at the Centre for Food Safety, University College Dublin. All the isolates were recovered during the period 2011–2016 from environmental and food samples from powdered infant formula plants in Europe. Isolates were resuscitated by placing one bead in 10 mL of tryptone soya broth (TSB, Sigma-Aldrich, Wicklow, Ireland) and incubated statically for 24 h at 37 °C. Afterward, a loop was taken from the culture and streaked out on Tryptone Soya Agar (TSA, Sigma-Aldrich, Ireland) for checking purity and obtaining single colonies.

### 2.2. DNA Extraction

Single colonies were re-suspended in TSB and cultured for 18 h at 37 °C. Subsequently, 1.8 mL of the pure cultures was used for DNA isolation with a DNeasy UltraClean Microbial Kit (Qiagen, Manchester, UK) following the manufacturer’s protocol. Quality and DNA concentrations were determined using a Qubit 2.0 fluorometer (Invitrogen, Waltham, MA, USA) and Nanodrop spectrophotometer (Nanodrop ND-1000, NanoDrop Technologies, Wilmington, DE, USA).

### 2.3. WGS and Bioinformatic Analysis

Genomic libraries were prepared using the NebNext Ultra^TM^ II FS DNA library prep Kit for Illumina. Genomic libraries were quality-assessed using the Tapestation 4200 (Agilent Technologies, Santa Clara, CA, USA) and concentration determined with a Qubit 2.0 Fluometer. WGS was carried out on the Illumina V3 (300-bp paired-end) reagent kit on the MiSeq platform (Illumina, Santa Clara, CA, USA). The quality of the reads was assessed using FastQC (version 0.11.8) [13]. Specifically, reads were filtered for length, while those with a Phred score less than 30 were discarded using FastP (v 0.23.1) [14]. De novo assembling was performed using SPAdes (v3.13.0) [15], and the quality of each of the assembled contigs was assessed using QUAST (version 5.0.2) [16]. To determine the underlying genomic population structure of the isolates, a core genome maximum likelihood phylogenetic tree clustered using different STs (Sequence Types) was also constructed with 96 *C. sakazakii* isolates publicly available in the NCBI. Assembled genomes (FASTA file) were uploaded to Parsnp (v1.7.4) [17]. The generated tree was subsequently visualized and elaborated using iToL (v4) [18]. Isolate ATCC BAA-894 was chosen as the reference isolate.

Multilocus sequence typing (MLST) was used to differentiate between the different strains according to seven housekeeping genes (*glnS*, *gltB*, *atpD*, *gyrB*, *ppsA*, *infB,* and *fusA*) using the MLST 1.8 tool available at “https://cge.cbs.dtu.dk/services/MLST/” (accessed on 6 April 2023).

### 2.4. Detection of Virulence and Antimicrobial Resistance Genes

The assemblies were annotated using Prokka (version 1.14.6), and the GFF files were delivered and were further analyzed with Roary version 3.13.0. [19,20]. Antibiotic resistance genes were detected from the assemblies and were compared with the Resistance Gene Identifier (RGI) tool in the Comprehensive Antibiotic Resistance Database (CARD) [21] and the ResFinder tool from the Center of Genomic Epidemiology (CGE). Visualization of the heatmaps showing the in silico-based detection of virulence-associated genes and AMR-encoding genes for all 18 *C. sakazakii* isolates were created using the R packages (version 4.2.2) pheatmap, ggplot2, tidyr, and dplyr.

### 2.5. Antimicrobial Susceptibility Test

The minimum inhibitory concentration (MIC) against 24 antibiotics (Cefazolin, Minocycline, Tigecycline, Ceftazidime, Ciprofloxacin, Ceftriaxone, Ceftaozolone/Tazobactam, Nitrofurantoin, Ceftazidime/Avibactam, Ampicillin/Sulbactam, Ampicillin, Amikacin, Aztreonam, Doripenem, Ertapenem, Cefepime, Gentamicin, Imipenem, Levofloxacin, Meropenem, Piperacillin/Tazobactam, Trimethoprim/Sulfamethoxazole, Tertacycline, and Tobramycin) was determined through the broth microdilution method using commercial Thermo Scientific™ Sensititer Susceptibility Plates (GN7F).(Serosep, Limercick, Ireland) Each plate was dosed with antimicrobial agents at appropriate dilutions. The results were read manually through visual reading of the growth in each well. The resistance/susceptibility profiles were expressed as sensitive (S), intermediate (I), and resistant (R), according to the European Committee on Antimicrobial Susceptibility Testing criteria 2022 (EUCAST 2022).

### 2.6. Detection of Plasmids and Prophages

The plasmid type was identified using the PlasmidFinder tool available at https://cge.cbs.dtu.dk/services/PlasmidFinfder/ (accessed on 18 January 2023) [22]. Prophage sequences were searched to verify the contribution of integrative elements in the structure of bacterial genomes and virulence factors; their sequences were predicted using the PHAge Search online Tool (PHASTER) [23]. According to the score assigned by the software (Intact: score > 90; Questionable: score 70–90; Incomplete: score < 70), the intact prophage regions were considered in this study. If the number of certain phages in this table was more than or equal to 100% of the total number of CDSs of the region, the region was marked with a total score of 150.

## 3. Results and Discussion

### 3.1. General Features of C. Sakazakii Strains

The 19 *C. sakazakii* isolates were whole-genome sequenced, and the draft assemblies ranged in size from 4.4 to 4.7 Mb and coding sequences (CDSs) comprised over 98% of the entire genome, with GC content ranging from 56.35 to 57.06% (Supplementary Table S1). MLST typing was used to find the genetic relatedness of the *C. sakazakii* isolates, and it contributed to our understanding of the genetic diversity and population structure of *C. sakazakii* isolates in our study. In total, 58% of the isolates belonged to ST 1 and the remainder to ST 4 (26%) and ST 99 (16%). ST 1 isolates were mostly recovered from environmental samples in the processing plant. However, raw material was also considered as a carrier of ST1 strains. *C. sakazakii* ST1 has been most frequently isolated from commercial PIF in various countries [1,2,3,4]. The main origin of ST 4 was curd and powder samples, while ST 99 was found in casein powder. A study in America showed that the majority of the isolated *C. sakazakii* strains recovered from PIF belonged to ST 1 and 4 [24].

### 3.2. Phylogenetic Analysis

The core genome phylogenetic analysis played a pivotal role in our study through combining genomic data with metadata to gain valuable insights into the epidemiological aspects of *Cronobacter* outbreaks. By examining the genetic relatedness of the isolates, we aimed to elucidate potential transmission patterns and trace the origins of these strains, which is crucial for understanding and controlling outbreaks in processing facilities.

**Figure 1:**
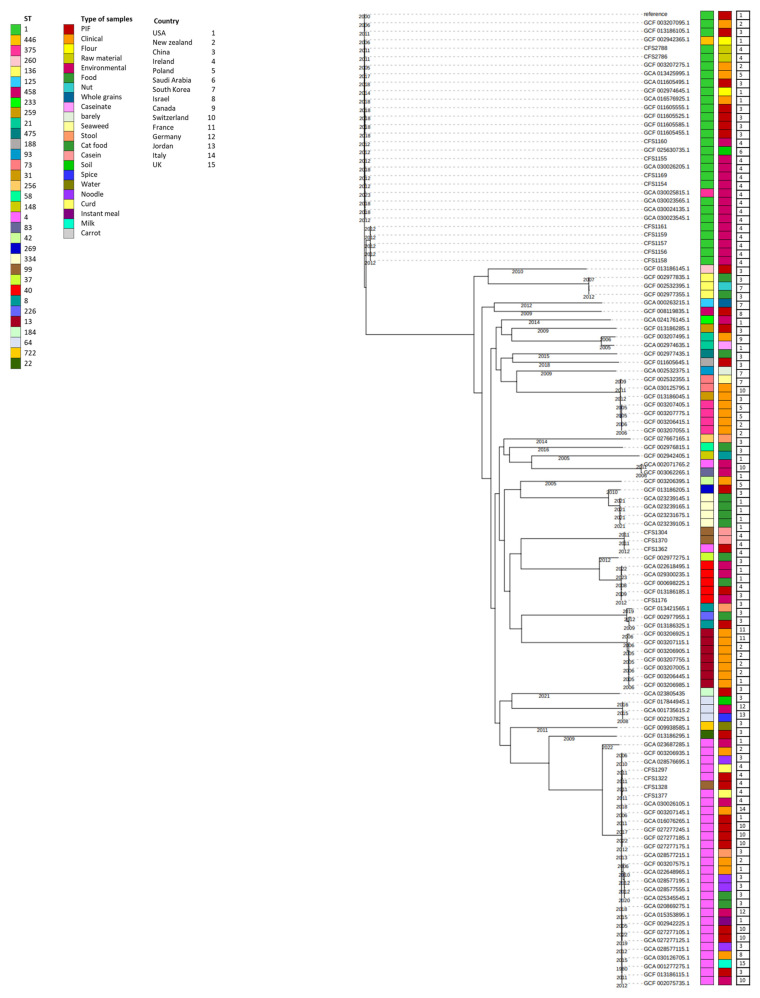
A maximum likelihood phylogeny for 18 *C. sakazakii* species showing their source associations. These were clustered according to their current phylogroups comparing the study isolates with 96 other genomes taken from the NCBI databases. Isolation information including sequence type and type of sample as well as the country of origin are shown with stripes in different colors and numbers, respectively. The years of isolation are shown on the phylogeny branches.

Furthermore, our research goes beyond the scope of a single setting. We aimed to contribute to the broader “One Health” perspective, considering the connections among different isolates recovered from various sources in different regions. Our analysis aimed to identify possible relationships and clues about the spread of *Cronobacter* strains across geographic boundaries, which has significant implications for public health. A phylogenetic tree was constructed to observe the clustering of the *C. sakazakii* study isolates (18 isolates) and to compare these with other previously characterized genomes available in the databases (96 isolates) and for which complete metadata are merged with the phylogenetic tree (Figure 1 and Supplementary Table S2). The maximum likelihood phylogenetic analysis reveals distinct clusters among the study isolates. Notably, one cluster predominantly comprises isolates with ST 1, including CFS2788, CFS2788, CFS2786, CFS1160, CFS1155, CFS1169, CFS1154, CFS1157, CFS1158, CFS1159, and CFS1161. Most of these isolates, along with other *C. sakazakii* database isolates of sequence type 1 (ST1), are primarily associated with environments related to powdered infant formula (PIF) and the raw materials used in PIF production. However, a limited number of ST1 isolates have been identified in clinical samples from Poland and New Zealand.

A separate cluster comprises CFS1304, CFS1370, and CFS1362 isolates, attributed to ST99 and ST4, respectively. These isolates are predominantly associated with casein and powdered infant formula (PIF) sources. All these isolates originated from Ireland.

Additionally, CFS1297, CFS1322, CFS1328, and CFS1377 are included in another, primarily comprising isolates with ST4, except for CFS1328, which belongs to ST99. The primary sources of ST4 isolates in this cluster are food items, particularly PIF and milk powder, although a few clinical and environmental samples were also identified.

### 3.3. Virulence Factors Identified in the Selected Isolates

**Figure 2:**
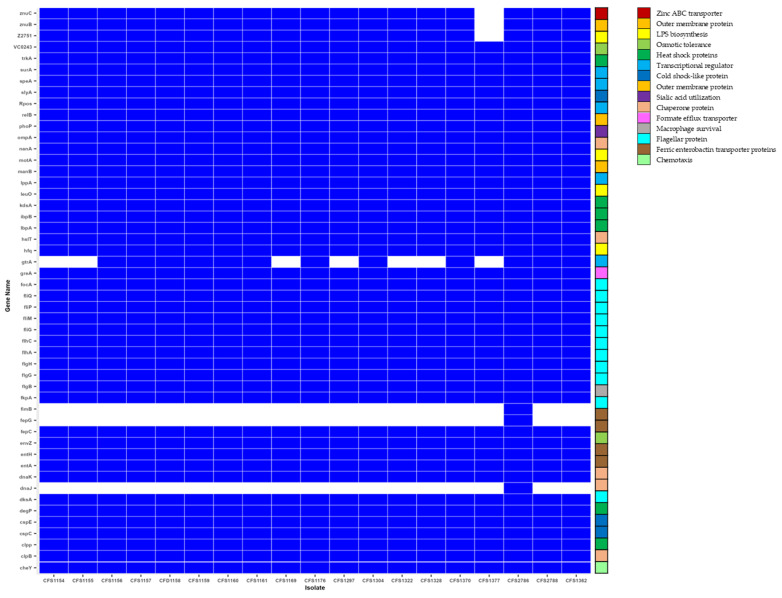
Heat map showing the profile of virulence factor (VF) genes for the 18 *C. sakazakii* isolates (white: sequence not detected; blue: sequence detected).

The molecular features of the virulence factors identified in the selected isolates are associated with environmental persistence, infection, and persistence in the host cell (Figure 2 and Supplementary Table S3).

#### 3.3.1. Virulence Factors Associated with Environmental Persistence

The genomic features related to the persistence of *C.sakazakii* in PIF plants are heat tolerance, osmotic and desiccation tolerance, and efflux systems supporting this persistence, such as potassium glutamate efflux and biofilm formation [6].

##### Adaptation to High Temperature

*Cronobacter* spp. are more heat-resistant compared to other *Enterobacteriaceae*. The genes encoding heat shock proteins including *hslT*, *ibpA*, *ibpB*, *degP*, and *clpP* were identified in all the selected isolated through WGS. A study performed by Zhao et al. [7] showed that the abundance of *IbpA* in *C. sakazakii* strains could significantly increase after heat shock. Different mechanisms could induce heat tolerance in *C. sakazakii* isolates following heat shock treatment such as an increased proportion of saturated fatty acids (SFAs), cyclic fatty acids (CFAs), and the ratio of saturated fatty acids in the cell membrane [8]. It has been reported that *hsIT* acts as a homologue of *ibpA* and *ibpB* in *Salmonella*. Transcription of *hsIT* could be increased after heat shock, leading to membrane permeability. This could eventually increase cell survival in harsh conditions such as high temperatures and desiccation [9,10]. Following heat shock or other stress conditions, *degP* acts as a periplasmic serine Endo protease, which degrades transiently denatured and unfolded proteins that accumulate in the periplasm. Its proteolytic activity is essential for the survival of cells at elevated temperatures [10]. Similar function is also attributed to *clpP. ClpP* is an ATP-dependent *Clp* protease proteolytic subunit that can degrade or refold damaged proteins in bacterial cells under stressed conditions such as high temperatures. This could repair proteins in the cell to protect them from further environmental stresses [11].

##### Adaptation to Acidic Environments, Osmotic Pressure, and Desiccation

The genes *cspC* and *cspE,* encoding cold shock proteins, were found in all *C. sakazakii* isolates. A study showed that the upregulation of cold shock proteins is mainly caused in stressed bacterial cells [25]. Furthermore, cold shock could increase the resistance to acidic environments [26]. The *speA* gene, which was also found in all study isolates, is rarely characterized in *C. sakazakii*, but has previously been reported as a ribosomal protein-coding genes, protecting bacterial cells against environmental challenges including high salt, osmotic stress, reactive oxygen species, and acidic environments [27]. *RpoS*, as a stress response sigma factor, is essential in regulating the response of *C. sakazakii* to osmotic stress [27,28,29]. This gene was also detected in the *C. sakazakii* isolates investigated using WGS in this study. The primary desiccation response involved the rapid accumulation of potassium glutamate to provide temporary protection against desiccation stress by immediately increasing the internal osmotic pressure of the bacterial cell.

The chaperone proteins *clpB*, *dnaK*, *hfq*, and *surA* were identified in all the isolates. Furthermore, another chaperone protein, *dnaJ*, was only found in the CFS2786 isolate. Chaperone proteins in *C. sakazakii* could be considered as virulence factors contributing to tolerance of cells under stressful conditions such as exposure to sanitizers, starvation, desiccation, and low pH [30]. Previous comparative transcriptome analysis revealed that *Clbp* has been associated with the pathogenicity of *C. sakazakii* [31] and was found in all the analyzed isolates. *hfq,* identified in all the investigated isolates, is one of the most important virulence factors conferring stress tolerance of *C. sakazakii* in PIF plants [32]. Other virulence factors found in all study isolates that contribute to environmental persistence include *trkA* and *leuO*. TrkA encodes a potassium uptake system [33] and some studies revealed that *trkA* was a resistant factor for *C. sakazakii* in osmotic and desiccation situations [33,34]. *LeuO* is a universal transcriptional activator affecting different cellular processes, including stress response [35].

#### 3.3.2. Virulence Factors Associated with Invasion and Persistence in the Host Cell

Different mechanisms are employed by *C. sakazakii* for cell invasion, including motility, colonization and translocation, secretion systems, and quorum sensing [6]. In addition, iron acquisition, sialic acid utilization, antimicrobial resistance (AMR), and immune system invasion are persistence mechanisms in *C. sakazakii* [6].

##### Cell Attachment and Invasion

1-Flagellar and chemotaxis proteins

Flagella-related genes including *fimB*, *flgB*, *flgG*, *flgH*, *flhA*, *flhC fliG*, *fliM*, *fliP,* and *fliQ* were identified in all the isolates. However, *fimB* was only identified in the CFS2786 isolate. In addition, genes associated with chemotaxis, including *cheY* and *motA,* were also identified. Flagella contribute to the infection of *Cronobacter spp*. through chemotaxis, adhesion to and invasion of host cells, and biofilm formation [6,31,36]. Furthermore, *envZ*, the gene encoding osmolarity sensory histidine kinase, as a virulence membranous protein, was also identified in the sequenced isolates. This gene influences bacterial resistance to desiccation, biofilm formation, and flagella motility [35,37].

2-Outer membrane proteins (OMPs)

The genes *ompA*, *lppA,* and *Z2751* were detected in all the isolates. However, *Z2751* was not detected is the CFS1377 isolate. Due to their exposure to the outside of the cell, outer membrane proteins may contribute to the translocation of virulence factors, as well as adhesion and cell motility [6]. *OmpA* has been reported as an important cellular adhesion factor in *Cronobacter* spp. to epithelial and endothelial cells of human and animal origin [5,28,36]. In addition, *ompA* has also been mentioned as a stress-response-encoding gene, especially osmotic stress [38].

3-Lipopolysaccharides (LPSs)

The lipopolysaccharide (LPS)-encoding genes *VC0243*, *kdsA*, *manB*, and *gtrA* were annotated in the study isolates. The first three genes were available in all the isolates. However, *gtrA* was lacking in isolates CFS1169, CFS1297, CFS1322, CFS1328, and CFS2786. LPSs, also known as O-antigen or endotoxins, are responsible for virulence in *Cronobacter* spp. and disrupt epithelial tight junctions [28]. In addition, LPSs reduce the permeability of the infant intestinal wall, leading to bacterial invasion and systemic infection. LPSs are heat-tolerant and can remain in the PIF after processing, leading to the presence of endotoxin in the end product [39].

4-Macrophage survival

*PhoP* is a transcriptional regulatory protein associated with several genes attributed to the pathogenicity of *Cronobacter* spp., including macrophage survival. In addition, this gene can contribute to the modification of membrane structure by changing lipid A in LPSs. These modifications enable the cell to resist the antimicrobial peptides [36,40]. The gene *fkpA* is an FKBP-type peptidyl-prolyl cis–trans isomerase, which is available in all the study isolates. These genes enable the cell to survive, persist, and multiply within macrophages secreted by the host immune system. Other studies also confirmed that *fkpA*, as a macrophage infectivity potentiator, is considered as a virulence factor in *Cronobacter* spp. [29,41]. Another transcriptional factor, *slyA*, was identified in all isolates and has been implicated in regulating the activity of several genes associated with bacterial virulence. The induction of hemolytic activity of bacterial cells and survival in macrophages have been attributed to this gene [42].

5-Anti-Toxins

*RelB* is an anti-toxin coding gene identified in all the isolates. Antitoxins are generally low-molecular-weight proteins that limit the toxin activity in the host cell by forming nontoxic toxin–antitoxin complexes (TA), shielding toxin targets, or specifically degrading toxin mRNAs [5]. A few reports have identified the possible presence of toxins associated with *C. sakazakii* [1].

6-Utilization of Sialic Acid

Sialic acid is found naturally in breast milk and is generally supplemented in PIF due to its importance in brain development. However, *C. sakazakii* is able to utilize sialic acid enhance its ability to cause systematic infections [36]. *NanA* has been identified as an antitoxin-encoding gene in *C. sakazakii* in multiple research works [4,5,38] and was also found in the current study.

7-Iron Acquisition System

Iron is considered an essential element for bacterial metabolism, survival, and pathogenicity. Iron acquisition systems involve the reduction of ferric iron (Fe^3+^), which is biologically inaccessible to bacteria, to ferrous iron (Fe^2+^) [6]. These systems enhance the growth of bacteria under iron-limited conditions in the human body and support their pathogenicity. Different iron acquisition systems have been explored in *Cronobacter* spp. so far, including a siderophore-mediated iron acquisition system (*iucABCD* and *iutA*) and ABC transport-mediated iron uptake [43]. In addition, a ferric dicitrate transport system was detected in clinical isolates of *C. sakazakii* and *C. malonaticus* [43]. This difference in iron acquisition mechanisms in *Cronobacter* spp. is dependent on the availability of iron in the environment [6,29]. In this study, *entA*, *fepC*, *fepG,* and *entH,* encoding ferric enterobactin transport proteins, were identified in all tested isolates, except for *fepG,* which was only detected in CFS2786. In a similar study carried out by Grim et al. [33], it was revealed that three different loci in the chromosome are responsible for encoding enterobactin-like siderophores in the tested *Cronobacter* spp. One of the locus comprised 10 genes: *entH*, *entA*, *entB*, *entE*, *entC*, *fepB*, *entS*, *fepD*, *fepG*, and *fepC* [43].

8-Zinc Acquisition System

Zinc is an important element for pathogenic bacteria in low-nutrient medium. Zinc acquisition is performed mainly by ATP binding cassette (ABC) transporters. Some periplasmic proteins or extracellular binding solute proteins are involved in the zinc acquisition system. They have high affinity to zinc and they can deliver it to the membrane permease for import into the cytoplasm [44]. In this study, *znuB* and *znuC* have been annotated as Zinc ABC transporter, permease protein and Zinc ABC transporter, ATP-binding protein, respectively. All the isolates possess these two Zinc ABC transporters except for CFS1377. It has been previously reported that zinc could enable *Cronobacter* spp. to survive in macrophages [36].

### 3.4. Antimicrobial Resistance Genes

**Figure 3:**
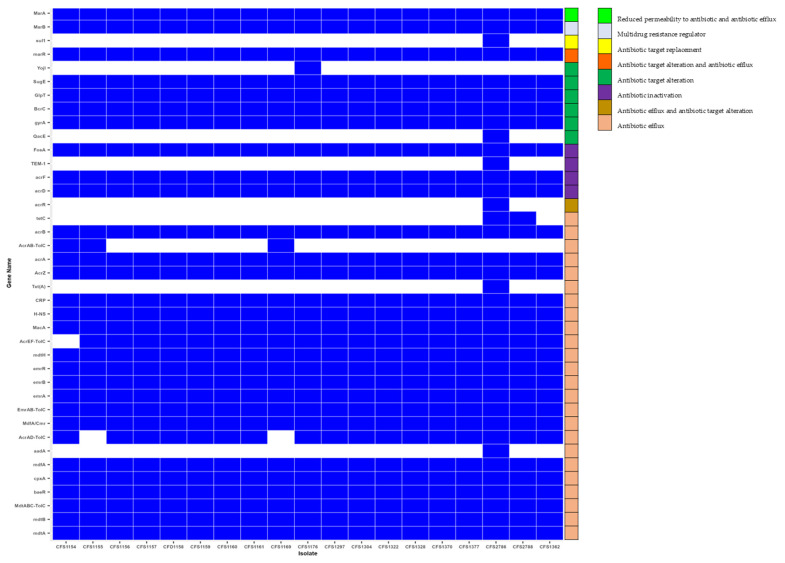
Heat map showing the profile of AMR genes for the 18 *C. sakazakii* isolates (white: sequence not detected; blue: sequence detected).

A total of 39 genes associated with antimicrobial resistance were predicted in the *C. sakazakii* isolates recovered from PIF production plants in this research (Supplementary Table S4 and Figure 3). In silico analysis of the AMR genes in the *C. sakazakii* isolates confirmed *acrA*, *AcrAB-TolC*, *AcrAD-TolC*, *acrB acrD AcrEF-TolC*, *acrF*, *acrR AcrZ*, *baeR*, *BcrC*, *cpxA*, *CRP*, *emrA*, *EmrAB-TolC*, *emrB*, *emrR*, *FosA*, *GlpT*, *gyrA*. *H-NS*, *MacA*, *MarA*, *MarB*, *marR*, *mdfA*, *MdfA*/*Cmr*, *mdtA*, *MdtABC-TolC*, *mdtB*, *mdtH*, *QacE*, and *SugE* in all isolates. These genes were previously identified in *C. sakazakii* strains investigated in other studies [45,46,47]. All the isolates had genes encoding resistance to at least one of the antibiotic classes such as Aminoglycoside, Fluoroquinolone, Macrolide, Aminocoumarin, Tetracycline, Microcin, peptide antibiotic, and phosphonic acid (*mdtA*, *mdtB*, *MdtABC-TolC*). The higher frequency of resistance in the studied AMR genes was observed in isolates treated with Cephalosporin, followed by Tetracycline and Penam (Penicillin) antimicrobial agents. These antibiotics are members of ß-lactam antibiotics. ß-lactamase activity in *C. sakazakii* isolates has been frequently reported [4,45,46,48].

In this study, multiple genes were predicted to induce resistance to a minimum of 2 and maximum of 11 antibiotic classes. Surprisingly, all the isolates carried *acrZ* and *marA* genes, associated with multidrug efflux pumps and transcriptional activators, respectively. In similar previous studies [49], *C. sakazakii* strains isolated from PIF plants exhibited resistance toward several antimicrobial compounds. The main mechanism of resistance of the tested isolates toward different antibiotics has been mostly dedicated to efflux pump systems. However, antibiotic target alteration and inactivation were assigned to *marR*, *marB*, *aadA BcrC*, *FosA*, *GlpT*, *sul1,* and *blaTEM-1* [49].

Unique AMR genes were predicted in CFS2786 and CFS2788 (ST1). These genes are *acrR*, *qacE*, *tet*(A), *tetC*, *yojI*, *sul1,* and *blaTEM-1*. *blaTEM-1* encodes resistance to multiple antibiotics (Penam/Penem/Cephalosporin/Monobactam), which was annotated in CFS2788.

### 3.5. Antibiotic Resistance Profile

Table 1 shows the resistance profile of the isolates to different tested antibiotics in in vitro conditions. In addition, the minimum inhibitory concentration (MIC) of each antibiotic is amended. In the current study, the results showed the isolates had sensitivity to a minimum of 16 and maximum of 22 antibiotics. According to previous studies, *Cronobacter* strains isolated from foods exhibited sensitivity to almost all the antibiotics used in in vitro conditions [4,5,50]. There was no correlation between ST type and susceptibility to different antibiotics. All the isolates exhibited resistance to at least two antibiotics, including cefazolin and minocycline. These two antibiotics belong to the drug class of cephalosporin and tetracycline, respectively.

In a study performed by Fei and Jiang [51], it was shown that all the *C. sakazakii* strains isolated from PIF and processing environments were resistant to Cefazolin. These results were in accordance with the in silico analysis of genes associated with AMR, since we found the highest frequency of AMR genes mediating resistance toward Cephalosporin, followed by Tetracycline.

Interestingly, all the isolates belonging to ST 1 recovered from a PIF factory only exhibited resistance to these two antibiotics. In total, 16% of the isolates (CFS1304 and CFS2788) exhibited resistance to three different antibiotics, including Ampicillin/Sulbactam (Penicillin) and Ciprofloxacin (Fluoroquinolones), respectively, apart from Cefazolin and Minocycline. CFS1322 isolates exhibited resistance to cephalosporins, including Cefazolin, minocycline, Ceftazidime, and Ceftriaxone. The CFS2788 isolate demonstrated resistance to several classes of antibiotics, including Cefazolin, Minocycline, Ampicillin (Penicillin), Ceftaozolone/Tazobactam (Cephalosporins), ciprofloxacin (fluoroquinolones), ceftazidime/avibactam (Cephalosporins), Nitrofurantoin, and Ampicillin/Sulbactam (Penicillin). This isolate was recovered from raw material in a PIF factory in Europe. According to the results, resistance to Cephalosporin is widespread among the tested isolates. Different studies correlated the cephalosporin resistance to potential ß-lactamase activity in *Cronobacter* strains and suggested the monitoring of ß-lactamase activity of the strains isolated from PIF [50,52].

### 3.6. Detection of Plasmids and Mobile Genetic Elements

Table 2 shows the intact plasmids found in the some of the studied *C. sakazakii* isolates. A single type of plasmid (Col(pHAD28) was identified in the isolates CFS1156, CFS1157, CFS1158, CFS1160, CFS1176 (ST 1), and CFS1362 (ST 4). All these isolates except CFS1362 originated from the same PIF processing plant in 2011. Holý, Parra-Flores [3] also found this plasmid in *C. sakazakii* strain 12683-2A isolated from powdered milk. In addition, this plasmid was also identified in *C. maloniticus* recovered from powdered infant formula in Chile [5]. In other studies, this plasmid has been associated with antibiotic resistance in *Klebsiella michiganensis* and *Salmonella enterica* subsp. *Enterica* [53,54].

The search for prophages in the studied genomes (Supplementary Table S5) showed that all the *C. sakazakii* isolates carried at least one intact phage. Prophage presence in the isolates could provide antibiotic and environmental stress resistance. In addition, it allows the bacteria to better adhere to the host cell and become pathogens [55]. Interestingly, all the detected phages except PHAGE_Aeromo_vB_AsaM_56_NC_019527(12) and PHAGE_Erwini_EtG_NC_047833(12) were identified in all the *C. sakazakii* strains isolated from various filth flies as reported by Jang, Chase [56]. These isolates were clustered among strains obtained from clinical, food, and environmental source isolates. In this study, 63.15%, including nine isolates from ST1, two isolates from ST 99, and one from ST 4, harbored the PHAGE_Salmon_118970_sal3_NC_031940 phage sequence. The length for this phage in the isolates varied between 110 and 150 kbp. This phage sequence was also identified in many *C. sakazakii* strains isolated from various filth flies [56]. In total, 32% of the isolates (ST 1 and ST 4) carried the PHAGE_Salmon_SSU5_NC_018843 phage. The length of the phage was identical among the isolates. This phage was also identified in *Pseudomonas* strains found in coastal water of Michigan state in the USA. It was reported that this phage could induce siderophore production and resistance to the heavy metals mercury and copper [57]. The PHAGE_Entero_Tyrion_NC phage was detected in isolates from ST1 (CFS1156, 57, 58, and 59). These isolates were recovered from the environmental sources in the same PIF manufacturing plant. The PHAGE_Cronob_phiES15_NC_018454 phage was only detected in CFS 1160. This phage has also been recovered from healthy skin from the forearm of a human male in Africa [58]. The PHAGE_Cronob_ESSI_2_NC_047854 phage was detected in CFS 1304, CFS 1362, and CFS 1370 with identical sequence length (31 kbp). Genomic analysis of *Citrobacter Portucalensis Sb-2* by Yu, Xie [59] revealed that different prophages, such as the PHAGE_Cronob_ESSI_2_NC_047854 phage, could promote metalloid resistance to the microorganism. The PHAGE_Aeromo_vB_AsaM_56_NC_019527 phage was identified in the isolates CFS 1322, CFS1328, and CFS1297 recovered from food samples including curd and milk powder from the same PIF factory.

## 4. Conclusions

In conclusion, we analyzed 18 *Cronobacter* isolates collected from various sources within PIF manufacturing plants (2011–2015). ST1 was the predominant sequence type, and all isolates carried plasmids and phages. Virulence factors suggested adaptability to environmental persistence and host cell infection. Notably, resistance to Cephalosporin was prevalent, followed by Tetracycline and Penicillin, irrespective of the source. All isolates showed in vitro resistance to at least two antibiotics, confirming genotypic–phenotypic correlation. The *Col(pHAD28)* plasmid was found in the isolates originating from the same source in the PIF factory. All isolates had intact phages. These data enhance our understanding of *C. sakazakii* behavior in PIF plants, aiding monitoring and control. Differentiated virulence and AMR traits inform targeted preventive measures for safer PIF consumption by vulnerable populations.

## Figures and Tables

**Table 1:** Antibiotic resistance profile of the 18 *C. sakazakii* study isolates.

Isolates	Antibiotics	MIC * (µg)	Antibiotic Resistance Profile
CFS1154	FAZ **	8	R ***
	MIN	4	R
CFS1155	MIN	4	R
CFS1156	FAZ	8	R
	MIN	2	R
CFS1157	FAZ	8	R
	MIN	4	R
CFS1158	FAZ	16	R
	MIN	4	R
CFS1159	FAZ	8	R
	MIN	2	R
CFS1160	FAZ	8	R
	MIN	2	R
CFS1161	FAZ	8	R
	MIN	2	R
CFS1169	FAZ	8	R
	MIN	2	R
CFS1176	FAZ	16	R
	MIN	2	R
	A/S 2	8/4	R
CFS1297	FAZ	4	R
	MIN	4	R
CFS1304	FAZ	16	R
	MIN	4	R
	CIP	2	R
CFS1322	FAZ	8	R
	MIN	4	R
	TAZ	2	I
	AXO	1	S
CFS1362	FAZ	8	R
	MIN	4	R
CFS1328	FAZ	8	R
	MIN	4	R
CFS1370	FAZ	8	R
	MIN	4	R
CFS1377	FAZ	8	R
	MIN	2	R
CFS2786	AMP	16	R
	FAZ	16	R
	C/T	4	R
	CIP	2	R
	MIN	4	R
	CZA	16/4	R
	NIT	64	R
	A/S 2	16/8	R
CFS2788	FAZ	8	R
	MIN	4	R
	CIP	2	R

* MIC (Minimum inhibitory concentration)—the lowest concentration of the antibiotics that can effectively inhibit growth of the tested microorganism. ** FAZ: Cefazolin, MIN: Minocycline, TGC: Tigecycline, TAZ: Ceftazidime, CIP: Ciprofloxacin, AXO: Ceftriaxone, C/T: Ceftaozolone/Tazobactam 4, NIT: Nitrofurantoin, CZA: Ceftazidime/Avibactam, A/S 2: Ampicillin/Sulbactam 2:1 ratio, AMP: Ampicillin. *** R: resistant; S: susceptible; I: intermediate resistance.

**Table 2:** Plasmids found in the *C. sakazakii* isolates with the use of the PlasmidFinder database.

Isolate	Plasmid	%Identity	Query/Template Length	Contig	Position in Contigs	Accession No.
CFS1156	Col(pHAD28)	94.96	119/131	NODE_38_length_3676_cov_857.909693	1716...1834	KU674895
CFS1157	Col(pHAD28)	94.96	119/131	NODE_41_length_3676_cov_857.909693	1716...1834	KU674895
CFS1158	Col(pHAD28)	94.96	119/131	NODE_41_length_3676_cov_857.909693	1716...1834	KU674895
CFS1160	Col(pHAD28)	94.96	119/131	NODE_37_length_3672_cov_866.208737	1610...1728	KU674895
CFS1176	Col(pHAD28)	94.96	119/131	NODE_106_length_2518_cov_463.535120	1949...2067	KU674895
CFS1362	Col(pHAD28)	94.96	119/131	NODE_30_length_3676_cov_5.826291	1705...1823	KU674895

## Data Availability

Data available in a publicly accessible repository. Accession numbers are provided in Table S1.

## Supplementary Materials

The following supporting information can be downloaded at: https://www.mdpi.com/article/10.3390/microorganisms11112749/s1. Supplementary Table S1: Feature of the sequenced genomes assembled with SPAdes; Supplementary Table S2. Metadata for study isolates and 96 isolates retrieved from the NCBI Database; Supplementary Table S3: Assignment of accession numbers to virulence genes in the study *C. sakazakii* isolates; Supplementary Table S4. Antibiotic resistance class patterns of *C. sakazakii* strains isolated from PIF processing plants. Table S5: Details of intact phage sequences identified using PHASTER software in the 18 *Cronobacter sakazakii* isolates’ genomes.
