# Supplementary material for: Comprehensive Genomic Characterization of Cronobacter sakazakii Isolates from Infant Formula Processing Facilities Using Whole-Genome Sequencing
Source: Microorganisms. 2023 Nov 11;11(11):2749. doi: 10.3390/microorganisms11112749 (PMC10673598; doi:10.3390/microorganisms11112749)
Supplement: Supplementary file 1 [file microorganisms-11-02749-s001.zip › Supplementary Table S1.pdf]

**Supplementary Table S1.** Feature of the sequenced draft genomes assembled with SPAdes and quality checked with Quast.

| Isolate | Total length (bp) | #Contigs | N50 | L50 | ST | G+C content (%) | CDSs (bp) | Accession number |
|---|---|---|---|---|---|---|---|---|
| CFS1154 | 4542213 | 50 | 199872 | 8 | 1 | 56.7 | 4446777 | JAWLKX000000000 |
| CFS1155 | 4556805 | 39 | 260217 | 7 | 1 | 56.69 | 4455968 | JAWLKW000000000 |
| CFS1156 | 4640775 | 61 | 232291 | 7 | 1 | 56.69 | 4509936 | JAWLSK000000000 |
| CFS1157 | 4641177 | 66 | 211383 | 8 | 1 | 56.7 | 4542177 | JAWLSJ000000000 |
| CFS1158 | 4612695 | 66 | 211383 | 8 | 1 | 56.7 | 4583034 | JAWLKV000000000 |
| CFS1159 | 4609363 | 65 | 195298 | 7 | 1 | 56.69 | 4547080 | JAWLKU000000000 |
| CFS1160 | 4542966 | 46 | 203518 | 7 | 1 | 56.76 | 4463582 | JAWLKT000000000 |
| CFS1161 | 4621986 | 361 | 230205 | 61 | 1 | 56.35 | 4612327 | JAWLSI000000000 |
| CFS1169 | 4572077 | 173 | 651609 | 24 | 1 | 56.6 | 4502144 | JAWLSH000000000 |
| CFS1297 | 4531883 | 44 | 166921 | 8 | 4 | 56.67 | 4504511 | JAWLKN000000000 |
| CFS1304 | 4420999 | 40 | 267914 | 7 | 99 | 57.05 | 4356816 | JAWLKO000000000 |
| CFS1322 | 4621902 | 51 | 265761 | 6 | 4 | 56.7 | 4564789 | JAWLKQ000000000 |
| CFS1328 | 4614795 | 56 | 224690 | 8 | 4 | 56.49 | 4609599 | JAWLKP000000000 |
| CFS1362 | 4603160 | 86 | 253680 | 7 | 99 | 56.76 | 4569007 | JAWLKM000000000 |
| CFS1370 | 4425465 | 20 | 601557 | 3 | 99 | 57.06 | 4225455 | JAWLKL000000000 |
| CFS1377 | 4508875 | 49 | 152844 | 9 | 4 | 56.69 | 4448610 | JAWLKK000000000 |
| CFS2786 | 4748603 | 212 | 222391 | 8 | 1 | 56.59 | 4698933 | JAWLKS000000000 |
| CFS2788 | 4652879 | 85 | 222391 | 8 | 1 | 56.6 | 4632748 | JAWLKR000000000 |
