# Supplementary material for: Comprehensive Genomic Characterization of Cronobacter sakazakii Isolates from Infant Formula Processing Facilities Using Whole-Genome Sequencing
Source: Microorganisms. 2023 Nov 11;11(11):2749. doi: 10.3390/microorganisms11112749 (PMC10673598; doi:10.3390/microorganisms11112749)
Supplement: Supplementary file 1 [file microorganisms-11-02749-s001.zip › Supplementary Table S2.pdf]

Supplementary Table S2. Metadata for Study Isolates and 96 Isolates Retrieved from the NCBI Database

Isolates	ST	Type of samples	Date of isolation	Country
**Reference**	1	PIF	2000	USA
**GCA_003207095.1**	1	Clinical	2006	New zealand
**GCA_013186105.1**	1	PIF	2011	China
**GCA_002942365.1**	446	flour	2006	USA
**CFS2788**	1	Raw material	2011	Ireland
**CFS2786**	1	Raw material	2011	Ireland
**GCA_003207275.1**	1	Clinical	2005	New zealand
**GCA_013425995.1**	1	Clinical	2017	Poland
**GCA_011605495.1**	1	PIF	2018	China
**GCA_002974645.1**	1	flour	2004	USA
**GCA_016576925.1**	1	Clinical	2018	USA
**GCA_011605555.1**	1	PIF	2018	China
**GCF_011605525.1**	1	PIF	2018	China
**GCA_011605585.1**	1	PIF	2018	China
**GCA_011605455.1**	1	PIF	2018	China
**CFS1160**	1	Environmental samples	2012	Ireland
**GCA_025630735.1**	1	soil	2012	Saudi Arabia
**CFS1155**	1	Environmental samples	2012	Ireland
**GCA_030026205.1**	1	PIF environment	2018	Ireland
**CFS1169**	1	Environmental samples	2012	Ireland
**CFS1154**	1	Environmental samples	2012	Ireland
**GCA_030025815.1**	375	PIF environment	2023	Ireland
**GCA_030023565.1**	1	PIF environment	2018	Ireland
**GCA_030024135.1**	1	PIF environment	2018	Ireland
**GCA_030023545.1**	1	PIF environment	2012	Ireland
**CFS1161**	1	Environmental samples	2012	Ireland
**CFS1159**	1	Environmental samples	2012	Ireland
**CFS1157**	1	Environmental samples	2012	Ireland
**CFS1156**	1	Environmental samples	2012	Ireland
**CFS1158**	1	Environmental samples	2012	Ireland
**GCF_013186145.1**	260	PIF	2010	China
**GCA_002977835.1**	136	food	2007	China
**GCF_002532395.1**	136	nut	2011	South Korea
**GCA_002977355.1**	136	food	2012	China

**GCA_000263215.1**	125	ground whole grains	2012	South Korea
**GCA_008119835.1**	458	PIF	2009	Israel
**GCA_024176145.1**	233	environmental sample	2014	USA
**GCA_013186285.1**	259	PIF	2009	China
**GCA_003207495.1**	21	Clinical	2006	Canada
**GCA_002974635.1**	21	sodium caseinate	2005	USA
**GCA_002977435.1**	475	food	2015	China
**GCA_011605645.1**	188	PIF	2018	China
**GCA_002532375.1**	93	barely	2009	South Korea
**GCA_002532355.1**	73	dried seaweed	2009	South Korea
**GCA_030125795.1**	73	Clinical	2011	Switzerland
**GCA_013186045.1**	31	Clinical	2012	China
**GCA_003207405.1**	375	Clinical	2005	Poland
**GCA_003207775.1**	375	Clinical	2005	Poland
**GCA_003206415.1**	375	Clinical	2006	New zealand
**GCA_003207055.1**	375	Clinical	2006	New zealand
**GCA_027667165.1**	256	stool	2014	China
**GCA_002976815.1**	58	food	2016	China
**GCA_002942405.1**	148	Carrot	2005	USA
**GCF_002071765.2**	4	environmental swab sponge powder infant formula manufacturing facility	2011	Switzerland
**GCA_003062265.1**	83	environmental	2006	USA
**GCA_003206395.1**	42	Clinical	2005	Poland
**GCA_013186205.1**	269	PIF	2010	China
**GCA_023239145.1**	334	cat food	2021	USA
**GCA_023239165.1**	334	cat food	2021	USA
**GCA_023231675.1**	334	cat food	2021	USA
**GCA_023239105.1**	334	cat food	2021	USA
**CFS1304**	99	Casein Powder	2011	Ireland
**CFS1370**	99	Casein powder	2011	Ireland
**CFS1362**	4	PIF	2011	Ireland
**GCA_002977275.1**	37	food	2012	China
**GCA_022618495.1**	40	environmental swab sponge	2022	USA
**GCA_029300235.1**	40	environmental swab sponge	2023	USA
**GCA_000698225.1**	40	swab from food processing facility	2008	Ireland
**GCA_013186185.1**	40	PIF	2009	China
**CFS1176**	40	Environmental samples	2012	Ireland

**GCA_013421565.1**	8	Stool	2019	China
**GCA_002977955.1**	226	food	2012	China
**GCA_013186325.1**	8	PIF	2009	China
**GCA_003206925.1**	13	Clinical	2006	France
**GCA_003207115.1**	13	Clinical	2006	France
**GCA_003206905.1**	13	Clinical	2005	New zealand
**GCA_003207755.1**	13	Clinical	2005	New zealand
**GCA_003207005.1**	13	Clinical	2006	New zealand
**GCA_003206445.1**	13	Clinical	2005	New zealand
**GCA_003206985.1**	13	Clinical	2006	USA
**GCA_023805435.1**	184	PIF	2021	China
**GCA_017844945.1**	64	Soil	2016	China
**GCA_001735615.2**	64	environmental	2015	Germany
**GCA_002107825.1**	64	Spice	2008	Jordan
**GCA_009938585.1**	722	water	2011	China
**GCA_013186295.1**	22	PIF	2009	China
**GCA_023687285.1**	4	environmental	2022	USA
**GCF_003206935.1**	4	Clinical	2006	New zealand
**GCA_028576695.1**	4	Noodle	2010	China
**CFS1297**	4	Curd	2011	Ireland
**CFS1322**	4	PIF	2011	Ireland
**CFS1328**	99	PIF	2011	Ireland
**CFS1377**	4	Curd	2011	Ireland
**GCA_030026105.1**	4	PIF environment	2018	Ireland
**GCA_003207145.1**	4	Clinical	2006	Italy
**GCA_016076265.1**	4	PIF	2011	USA
**GCA_027277245.1**	4	PIF	2017	Switzerland
**GCA_027277185.1**	4	PIF	2022	Switzerland
**GCF_027277175.1**	4	PIF	2012	Switzerland
**GCA_028577215.1**	4	Stool	2013	China
**GCA_003207575.1**	4	Clinical	2006	New zealand
**GCA_022648965.1**	4	Clinical	2010	USA
**GCA_028577195.1**	4	Noodle	2012	China
**GCA_028577555.1**	4	Noodle	2012	China
**GCF_025345545.1**	4	food	2020	China

**GCA_020869275.1**	4	food	2018	China
**GCA_015353895.1**	4	environmental swab sponge powder infant formula manufacturing facility	2015	Germany
**GCA_002942225.1**	4	instant oatmeal with strawberries and cream	2005	USA
**GCA_027277105.1**	4	PIF	2022	Switzerland
**GCA_027277125.1**	4	PIF	2019	Switzerland
**GCA_028577115.1**	4	Rice Noodle of Infant	2012	China
**GCA_030126705.1**	4	Clinical	2015	Israel
**GCA_001277275.1**	4	Milk	1980	UK
**GCA_013186115.1**	4	PIF	2011	China
**GCA_002075735.1**	4	environmental swab sponge powder infant formula manufacturing facility	2012	Switzerland
