# Supplementary material for: Comprehensive Genomic Characterization of Cronobacter sakazakii Isolates from Infant Formula Processing Facilities Using Whole-Genome Sequencing
Source: Microorganisms. 2023 Nov 11;11(11):2749. doi: 10.3390/microorganisms11112749 (PMC10673598; doi:10.3390/microorganisms11112749)
Supplement: Supplementary file 1 [file microorganisms-11-02749-s001.zip › Supplementary Table S3.pdf]

Supplementary Table S3: Assignment of accession numbers to virulence genes in the study *C. sakazakii* isolates

Virulence genes	Accession number
cheY	Q93P00
cheZ	P0A9H9
clpB	Q47101
clpp	NP_465991
cspE	P80434
degP	P0C0V1
dksA	P18274
dnaJ	Q60004
dnaK	P17821
entA	Q2MBK7
entH	Q2MBK4
envZ	P41406
fepC	P75724
fepG	J5VAW4
fimB	P04742
fkpA	O08437
flgB	P0ABW9
flgG	P0ABX5
flgH	B5FGS5
flhA	P35620
flhc	O86047
fliG	A0A7U7FLF7
fliM	P06974
fliP	P54700
fliQ	P35535
focA	P0AC23
greA	P0A6W5
grtA	A0A0H2ZDG5
hfq	P0A6X3
hsIT	A0A0T9LQV8
ibpA	Q6CYV2
IppA	Q2LAM2
kdsA	A0A8E1QP66
leuO	P10151
manB	Q5B833
motA	Q53174
nanA	P62576
ompA	P24017
phoP	P23836
relB	P0C079
Rpos	P13445
slyA	P40676
speA	P21170
surA	P0ABZ6
trKA	P0AGI8
Vc0243	L0GAG6
