# Supplementary material for: Comprehensive Genomic Characterization of Cronobacter sakazakii Isolates from Infant Formula Processing Facilities Using Whole-Genome Sequencing
Source: Microorganisms. 2023 Nov 11;11(11):2749. doi: 10.3390/microorganisms11112749 (PMC10673598; doi:10.3390/microorganisms11112749)
Supplement: Supplementary file 1 [file microorganisms-11-02749-s001.zip › Supplementary Table S4.pdf]

Supplementary Table S4. Antibiotic resistance class patterns of *C. sakazakii* strains isolated from PIF processing plants.

Gene name	Resistance Patterns	Numbers class of antibiotics	No of isolates
acrA	Tetracycline / Phenicols/cephalosporin/rifamycin /disinfecting agents and antiseptics,/penam,/glycylcycline/fluoroquinolon		n=19
acrB	Tetracycline/rifamycin/glycylcycline/penam/phenicol/cephalosporin/disinfecting agents and antiseptics/fluoroquinolone		n=19
acrR	Tetracycline/glycylcycline/phenicol /cephalosporin/disinfecting agents and antiseptics/rifamycin/penam/fluoroquinolone	n=8	n=1
AcrAB-TolC	Tetracycline/phenicol/rifamycin/cephalosporin/glycylcycline/penam/disinfecting agents and antiseptics/fluoroquinolone antibiotic		n=3
marR	Tetracycline /glycylcycline/disinfecting agents and antiseptics/phenicol antibiotic/penam/rifamycin antibiotic/cephalosporin/fluoroquinolone		n=19
AcrAD-TolC	Aminoglycoside		n=17
acrD	Aminoglycoside		n=19
aadA	Aminoglycoside		n=1
emrA	Fluoroquinolone		n=19
emrB	Fluoroquinolone		n=19
emrR	Fluoroquinolone		n=19
gyrA	Fluoroquinolone		n=19
MarB	Fluoroquinolone		n=19
EmrAB-TolC	Fluoroquinolone		n=19
MacA	Macrolide antibiotic		n=19
mdtA	Aminocoumarin antibiotic		n=19
mdtB	Aminocoumarin antibiotic	n=1	n=19
mdtH	Aminocoumarin antibiotic		n=19
MdtABC-TolC	Aminocoumarin antibiotic		n=19
QacE	disinfecting agents and antiseptics		n=1
SugE	quaternary ammonium compounds		n=19
Tet(A)	Tetracycline		n=1
tetC	Tetracycline		n=2
YojI	Microcin(peptide antibiotic)		n=1
BcrC	bacitracin(peptide antibiotic)		n=19
FosA	phosphonic acid		n=19
GlpT	phosphonic acid		n=19
sul1	sulfonamide antibiotic		n=1
AcrEF-TolC	Fluoroquinolone/penam/cephamycin/cephalosporin		n=18
acrF	Fluoroquinolone/penam/cephamycin/cephalosporin	n=4	n=19
TEM-1	Penam/penem/cephalosporin/monobactam		n=1
AcrZ	Tetracycline/penam/disinfecting agents and antiseptics/ glycylcycline/rifamycin/phenicol/carbapenem/cephalosporin/monobactam/cepha-mycin/fluoroquinolone antibiotic	n=11	n=19
MarA			n=19

	Tetracycline/penam/disinfecting agents and antiseptics/ glycylcycline/rifamycin/phenicol /carbapenem/ cephalosporin/monobactam/cephamycin/fluoroquinolone antibiotic		
baeR	Aminocoumarin/Aminoglycoside		n=19
cpxA	Aminocoumarin/Aminoglycoside		n=19
CRP	Macrolide/ Fluoroquinolones	n=2	n=19
mdfA	Aminoglycoside/Cephalosporin		n=19
MdfA/Cmr	Disinfecting agents and antiseptics/Tetracycline		n=19
H-NS	Macrolide/Tetracycline/penam/cephalosporin/fluoroquinolone/cephamyc-in	n=6	n=19
