# Supplementary material for: Comprehensive Genomic Characterization of Cronobacter sakazakii Isolates from Infant Formula Processing Facilities Using Whole-Genome Sequencing
Source: Microorganisms. 2023 Nov 11;11(11):2749. doi: 10.3390/microorganisms11112749 (PMC10673598; doi:10.3390/microorganisms11112749)
Supplement: Supplementary file 1 [file microorganisms-11-02749-s001.zip › Supplementary Table S5.pdf]

Supplementary Table S5. Details of intact Phage sequences identified by PHASTER software in the 18 *Cronobacter sakazakii* isolates genome.

solate	Region	Lenght	Completeness	Score	Total proteins	Region Position	Most common phage	GC%
CFS1154	1	34.6Kb	intact	130	40	82112-116797	PHAGE_Salmon_118970_sal3_NC_031940(11)	50.74%
	1	34.4Kb	intact	110	39	81816-116313	PHAGE_Salmon_118970_sal3_NC_031940(10)	50.54%
CFS1155	4	102Kb	intact	150	133	1-102072	PHAGE_Salmon_SSU5_NC_018843(91)	51.15%
	1	46Kb	intact	150	63	249680-295734	PHAGE_Salmon_118970_sal3_NC_031940(10)	55.03%
CFS1156	4	42.4Kb	intact	98	56	89733-132186	PHAGE_Entero_Tyrion_NC_031077(34)	54.91%
	1	46Kb	intact	150	65	354841-400895	PHAGE_Salmon_118970_sal3_NC_031940(10)	55.03%
CFS1157	3	42.4Kb	intact	98	56	89733-132186	PHAGE_Entero_Tyrion_NC_031077(34)	54.91%
CFS1158	1	46Kb	intact	150	63	216579-262633	PHAGE_Salmon_118970_sal3_NC_031940(10)	55.03%
	5	42.4Kb	intact	98	56	89805-132258	PHAGE_Entero_Tyrion_NC_031077(34)	54.91%
	1	66.8Kb	intact	150	66	8964-75809	PHAGE_Salmon_118970_sal3_NC_031940(10)	56.27%
CFS1159	2	42.4Kb	intact	98	56	89733-132186	PHAGE_Entero_Tyrion_NC_031077(34)	54.91%
	1	47.8Kb	intact	150	42	381268-429096	PHAGE_Cronob_phiES15_NC_018454(9)	52.29%
CFS1160	3	44.4Kb	intact	140	40	119-44576	PHAGE_Salmon_118970_sal3_NC_031940(13)	54.49%
CFS1161	7	16.7Kb	intact	130	21	97-16887	PHAGE_Entero_mEp235_NC_019708(7)	55.17%
CFS1169	-	-	-	-	-	-	-	-
CFS1297	3	102.5Kb	intact	150	125	715-103309	PHAGE_Salmon_SSU5_NC_018843(92)	50.92%
	5	48Kb	intact	150	60	59-48139	PHAGE_Aeromo_vB_AsaM_56_NC_019527(12)	53.11%
	2	26.5Kb	intact	120	38	93876-120460	PHAGE_Salmon_118970_sal3_NC_031940(17)	53.55%
CFS1304	4	31Kb	intact	94	37	114061-145080	PHAGE_Cronob_ESSI_2_NC_047854(27)	53.37%
	6	32.4Kb	intact	150	38	28856-61310	PHAGE_Escher_pro483_NC_028943(12)	54.59%
CFS1322	1	81.8Kb	intact	150	99	15814-97667	PHAGE_Salmon_SSU5_NC_018843(75)	51.76%
	2	50.2Kb	intact	150	58	11006-612991	PHAGE_Aeromo_vB_AsaM_56_NC_019527(12)	53.46%

CFS1328	3	104.3Kb	intact	150	128	3-104381	PHAGE_Salmon_SSU5_NC_018843(92)	50.86%
	8	22.9Kb	intact	100	38	20-22960	PHAGE_Aeromo_vB_AsaM_56_NC_019527(11)	51.85%
CFS1362	3	31Kb	intact	94	34	67169-98188	PHAGE_Cronob_ESSI_2_NC_047854(27)	53.37%
	4	46.9Kb	intact	150	55	481566-128472	PHAGE_Salmon_118970_sal3_NC_031940(18)	51.71%
	5	32.4Kb	intact	150	40	43618-76072	PHAGE_Erwini_EtG_NC_047833(12)	54.58%
CFS1370	2	31Kb	intact	94	37	610239-641258	PHAGE_Cronob_ESSI_2_NC_047854(27)	53.37%
	4	38.3Kb	intact	150	48	413613-451964	PHAGE_Salmon_118970_sal3_NC_031940(18)	50.66%
CFS1377	3	100.1Kb	intact	150	127	4422-104541	PHAGE_Salmon_SSU5_NC_018843(92)	50.63%
CFS2786	1	34.6Kb	intact	130	40	81816-116501	PHAGE_Salmon_118970_sal3_NC_031940(11)	50.74%
CFS2788	1	34.4Kb	intact	110	39	81816-116313	PHAGE_Salmon_118970_sal3_NC_031940(10)	50.54%
	4	101.9Kb	intact	150	130	137-102074	PHAGE_Salmon_SSU5_NC_018843(95)	51.15%
